# ANCA-Associated Vasculitis Co-Occurrence With Systemic Sclerosis: A Case Report of a Rare Diagnostic Dilemma

**DOI:** 10.1177/2324709618785188

**Published:** 2018-06-28

**Authors:** Jordana Cheta, Michael Binder, Jolanta Kowalewska, Sandeep Magoon

**Affiliations:** 1 Eastern Virginia Medical School, Norfolk, VA, USA

**Keywords:** SRC, scleroderma, scleroderma renal crisis, MPO, ANCA-associated vasculitis, acute kidney injury, AKI, MCTD

## Abstract

Systemic sclerosis (SSc) is a rare autoimmune disorder that is typically divided into limited cutaneous systemic sclerosis and diffuse cutaneous systemic sclerosis. Scleroderma renal crisis (SRC) is a severe complication of SSc and typically presents with new-onset hypertension and a reduction in renal functioning. In patients presenting with typical features of SRC, treatment with an angiotensin-converting enzyme inhibitor along with dialysis as needed is typically initiated empirically. Renal biopsy is not recommended in patients with SSc presenting with typical features of SRC. Antineutrophil cytoplasmic antibody (ANCA)-associated vasculitis (AAV) is a rare co-occurrence with SSc, in around 2.5% to 9% of patients. AAV is an inflammatory condition that can result in renal failure due to mononuclear cell infiltration and destruction of blood vessels. Treatment of AAV is drastically different from SRC and typically consists of immunosuppressants and dialysis if needed. SRC and AAV can only reliably be distinguished by renal biopsy. We present a rare case of a 70-year-old female with limited cutaneous systemic sclerosis who presented to the emergency department with new-onset renal failure. Her serology was found to be positive for antinuclear antibodies and myeloperoxidase antibodies, resulting in a renal biopsy, which revealed an acute necrotizing vasculitis consistent with AAV. We suggest consideration of a renal biopsy in patients with SSc who present with new-onset renal failure, especially with nonresponse to SRC treatment or positive serology.

## Introduction

Systemic sclerosis (SSc) is an autoimmune disorder that results in inflammation and fibrosis of the skin, almost always, in addition to multiple other organs.^1^ It is classified into 2 subtypes based on the amount of skin involvement, limited cutaneous systemic sclerosis(lcSSc), which involves the hands, face, feet and forearms; and diffuse cutaneous systemic sclerosis (dcSSc), which involves the trunk and visceral organs typically.^1,2^ Scleroderma renal crisis (SRC) is one of the most severe complications of SSc, affecting 5% to 10% of SSc patients, with increased frequency in patients with dcSSc.^3,4^ The mechanism of SRC is still under investigation, but likely involves endothelial injury resulting in intimal thickening of renal interlobular and arcuate arteries.^4^ Arterial narrowing results in decreased renal perfusion and secondary hyperplasia of the juxtaglomerular apparatus, and an increase in activation of the renin-angiotensin-aldosterone axis, as well as upregulation of the endothelin axis.^4,5^ An additional trigger, possibly dehydration or nephrotoxic drug use, is typically the “second hit” associated with acute onset of SRC.^3,4^

Antineutrophil cytoplasmic antibody (ANCA)-associated vasculitis (AAV) is a rare co-occurrence in patients with SSc, around 2.5% to 9%, although the incidences are higher than occur in the general population and may suggest possibility of an overlap syndrome.^6,7^ Antibodies in AAV may be directed against myeloperoxidase (MPO), and stain in a perinuclear pattern (p-ANCA) on immunofluorescence, or directed against proteinase-3 (PR-3), and stain in a cytoplasmic pattern (c-ANCA).^8^ Antibodies against PR-3 are predominant in the United States of America and Europe, around 80%, whereas MPO antibodies are predominant in Asian countries.^8^ AAV, as compared with SRC, causes renal failure due to mononuclear cell infiltrate and destruction of the vessel wall.^9^ The 2 conditions can only reliably be distinguished by biopsy.^9^

Diagnostic challenges arise with acute kidney injury in patients with SSc, as SRC, AAV, and mixed connective tissue disease have markedly different treatment options, and a prompt diagnosis is crucial to optimize patient outcomes.^10,11^

We present a case of a 70-year-old female with SSc who presented with acute kidney injury and clinical symptoms suggestive of SRC but was found to have AAV.

## Case Report

We present the case of a 70-year-old female who was sent to the hospital by her family physician for an elevated blood urea nitrogen of 84 g/dL and a creatinine of 6.1 mg/dL. Baseline values were normal 1 month prior. Her chief complaints were weakness, decreased appetite, bilateral lower extremity swelling, and discoloration for the past 3 weeks. She has a past medical history significant for SSc, diagnosed in 1980, Raynaud’s disease, hypertension, and neuropathy. Of note, she was recently started on mycophenolate mofetil at a dose of 500 mg twice daily for treatment of her SSc.

**Figure 1.**
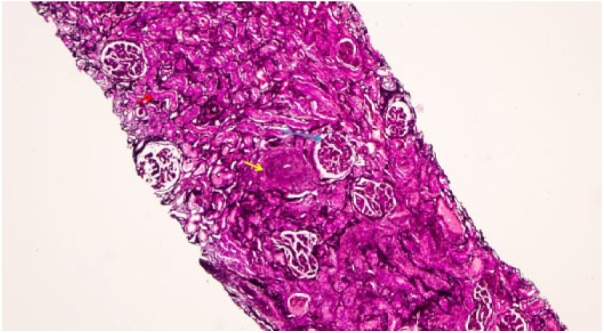
Renal cortex containing glomeruli with ischemic-type wrinkling of capillary walls (blue arrow). There is a background of diffuse interstitial fibrosis and tubular atrophy (red arrow). Arteries show prominent thickening due to concentric smooth muscle hyperplasia (yellow arrow;. Jones methenamine silver stain, original magnification 10×).

**Figure 2.**
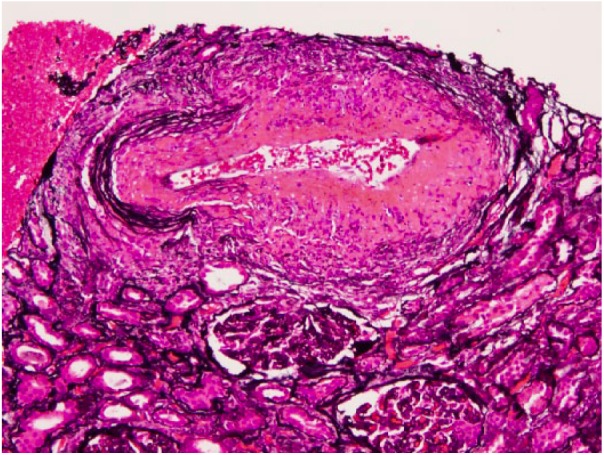
Artery with necrotizing vasculitis, with prominent transmural necrosis and inflammatory infiltrate (Jones methenamine silver stain, original magnification 20×).

**Figure 3.**
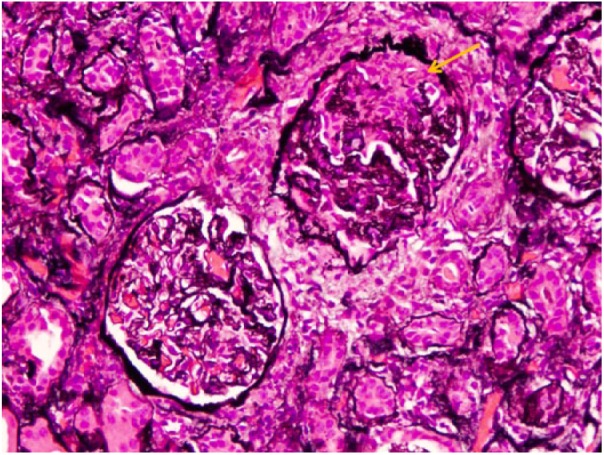
Crescent formation (yellow arrow; Jones methenamine silver stain, original magnification 20×).

On physical examination, she was hypertensive to 164/72 mm Hg, had bilateral lower extremity edema, and skin changes limited to her hands and feet, consistent with lcSSc. Her urinalysis was positive for proteinuria and hematuria. Her complete blood count was significant for decreased hemoglobin of 8.6 g/dL. Her serologies were positive for ANA at 1:160, and MPO antibodies, at a level of 23.8 by ELISA (enzyme-linked immunosorbent assay). PR-3 antibodies, p-ANCA, and c-ANCA were negative. Renal biopsy demonstrated an acute necrotizing vasculitis superimposed on chronic changes related to her SSc. Light microscopy demonstrated 28 to 38 glomeruli of which 10% were globally sclerosed. The majority of the remaining glomeruli show prominent ischemic-type wrinkling of capillary walls (Figure 1). Necrotizing vasculitis of the artery is seen, with prominent transmural necrosis and inflammatory infiltrate with prominent thickening of surrounding arterioles due to concentric hyperplasia (Figure 2). Ten percent of glomeruli per section show crescents (Figures 3).

Electron microscopy confirmed the absence of immune complexes as well as tubular epithelial necrosis and diffuse foot process effacement. Treatment was initiated with 8 sessions of plasma exchange with albumin, pulse steroids at a dose of 500 mg intravenous for 3 days, and rituximab at 1 g for 2 doses 2 weeks apart. The patient unfortunately remains hemodialysis dependent.

## Discussion

Several reviews of the literature have been composed for patients with SSc and AAV. Arad et al reviewed 40 cases of SSc and AAV in 2011 and reported that SRC typically occurs early in the course of SSc, with AAV occurring after several years of illness.^12^ In addition, hypertension, microangiopathic hemolytic anemia, and thrombocytopenia only occur in a minority of cases of AAV.^12^ In severe cases of AAV with SSc, high-dose corticosteroids and immunosuppression are utilized, with rituximab considered for resistant cases.^12^ Most cases of ANCA-associated glomerulonephritis in patients with SSc have been reported as “normotensive renal failure,” although we present a patient with clinical features for SRC (who did not have SRC) and was found to have AAV on serology and renal biopsy.^10,13,14^ Although high-dose corticosteroids (15 mg/kg/day) can contribute to the development of SRC in patients with SSc, via inhibition of prostacyclin production, which increases angiotensin-converting enzyme activity, it did not contribute in our patient.

Clinically, patients with SRC present with new-onset hypertension, >150/85 mm Hg, and decreased renal functioning, ≥30% reduction in estimated glomerular filtration rate.^4^ Patients may also exhibit headaches, oliguria or anuria, hypertensive encephalopathy, seizures, pulmonary edema, and visual disturbances.^4^ Microangiopathic hemolytic anemia, thrombocytopenia, elevated creatinine, and mild hematuria and proteinuria may also be present on laboratory evaluation.^4^ A worse prognosis for SRC exists in patients with dcSSc, cardiac involvement, renal crisis with normotensive blood pressures, and skin scores ≥20.^15^

Treatment of typical SRC relies on gradual lowering of blood pressure, usually by 20 mm Hg systolic per day, with angiotensin-converting enzyme inhibitors remaining the first-line medical treatment and additional antihypertensive agents included as needed.^1,4^ Dialysis is frequently required in patients with SRC, and is temporary for about half of patients.^3,4^ Importantly, biopsy is not typically required in patients who present with SRC with classical features.^3,16^ AAV, on the other hand, requires a tissue diagnosis and is typically treated with cyclophosphamide induction, followed by maintenance therapy with azathioprine, methotrexate, or mycophenolate, with 90% to 94% remission.^9^ Our case emphasizes the importance of a renal biopsy in a patient with SSc who presents with new-onset acute kidney injury due to the treatment for AAV being a time sensitive matter to prevent extensive permanent renal injury.

## Conclusion

This diagnostic dilemma argues for consideration of a biopsy in patients with SSc who present with new-onset renal failure, even if typical features exist for a diagnosis of SRC due to the risk of inappropriate therapy, which can result in permanent renal failure.
